# Radiomics combined with clinical and MRI features may provide preoperative evaluation of suboptimal debulking surgery for serous ovarian carcinoma

**DOI:** 10.1007/s00261-024-04343-3

**Published:** 2024-07-14

**Authors:** Li Liu, Wenfei Zhang, Yudong Wang, Jiangfen Wu, Qianrui Fan, Weidao Chen, Linyi Zhou, Juncai Li, Yongmei Li

**Affiliations:** 1 Department of Radiology, The People’s Hospital of Yubei District of Chongqing City, No. 23 ZhongyangGongyuanBei Road, Yubei District, Chongqing, 401120 China; 2 Department of Radiology, The First Affiliated Hospital of Chongqing Medical University, No. 1 Youyi Road, Yuanjiagang, Yuzhong District, Chongqing, 400016 China; 3 Institute of Clinical Algorithms, InferVision, Ocean International Center, Chaoyang District, Beijing, 100020 China; 4 Department of Radiology, Daping Hospital, Army Medical Center, Army Medical University, 10# Changjiangzhilu, Chongqing, 40024 China; 5 Department of Surgery, The People’s Hospital of Yubei District of Chongqing City, No. 23 ZhongyangGongyuanBei Road, Yubei District, Chongqing, 401120 China

**Keywords:** Suboptimal debulking surgery, Serous ovarian carcinoma, Radiomics, Predictive model, MRI

## Abstract

**Purpose:**

To develop and validate a model for predicting suboptimal debulking surgery (SDS) of serous ovarian carcinoma (SOC) using radiomics method, clinical and MRI features.

**Methods:**

228 patients eligible from institution A (randomly divided into the training and internal validation cohorts) and 45 patients from institution B (external validation cohort) were collected and retrospectively analyzed. All patients underwent abdominal pelvic enhanced MRI scan, including T2-weighted imaging fat-suppressed fast spin-echo (T2FSE), T1-weighted dual-echo magnetic resonance imaging (T1DEI), diffusion weighted imaging (DWI), and T1 with contrast enhancement (T1CE). We extracted, selected and eliminated highly correlated radiomic features for each sequence. Then, Radiomic models were made by each single sequence, dual-sequence (T1CE + T2FSE), and all-sequence, respectively. Univariate and multivariate analyses were performed to screen the clinical and MRI independent predictors. The radiomic model with the highest area under the curve (AUC) was used to combine the independent predictors as a combined model.

**Results:**

The optimal radiomic model was based on dual sequences (T2FSE + T1CE) among the five radiomic models (AUC = 0.720, P < 0.05). Serum carbohydrate antigen 125, the relationship between sigmoid colon/rectum and ovarian mass or mass implanted in Douglas’ pouch, diaphragm nodules, and peritoneum/mesentery nodules were considered independent predictors. The AUC of the radiomic–clinical–radiological model was higher than either the optimal radiomic model or the clinical–radiological model in the training cohort (AUC = 0.908 vs. 0.720/0.854).

**Conclusions:**

The radiomic–clinical–radiological model has an overall algorithm reproducibility and may help create individualized treatment programs and improve the prognosis of patients with SOC.

**Graphical abstract:**

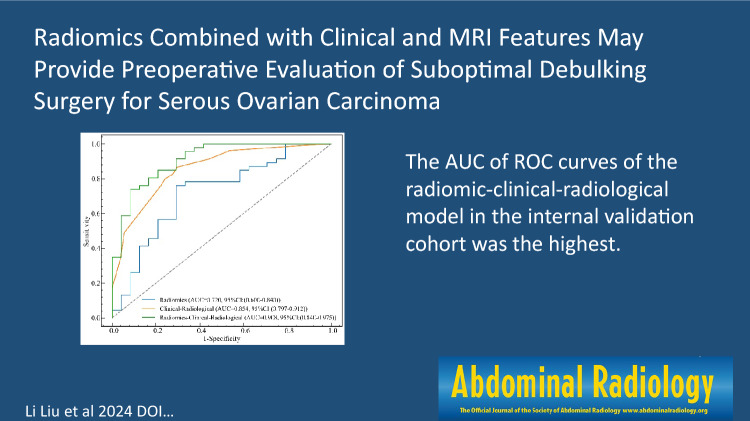

## Introduction

Serous ovarian carcinoma (SOC) is the most common histological type of ovarian carcinoma (OC) and has the highest mortality rate among gynecological tumors [1–4]. The resection of visible cancer lesions as more thoroughly as possible can prolong the survival of patients with SOC [5–7]. At present, primary debulking surgery (PDS) or neoadjuvant chemotherapy (NACT) followed by interval debulking surgery (IDS) is the standard of the clinical treatment for SOC. However, controversies exist about which one is better for patients with advanced ovarian cancer. Although NACT followed by IDS has a greater chance of achieving complete resection more easily in patients with a high disease burden, it does not necessarily lead to “more satisfactory” overall survival [4, 8]. Rauh-Hain et al. [9] demonstrated that NACT increases the risk of platinum resistance.

Optimal debulking surgery (ODS) was defined as the absence of residual disease (R0) after surgery. When R0 cannot be achieved, R1 (a residual disease with a maximum diameter of < 1 cm) is considered the next best option. To achieve ODS as much as possible, accurate preoperative assessment is essential. Nevertheless, this remains an arduous task. Lesions are extensively distributed in the abdominal cavity, and ascites usually leads to anorexia and poor physical condition. These factors complicate the physical condition of patients with SOC, which often increase the difficulty for gynecologists to evaluate surgical risks, outcomes, and prognosis preoperatively.

In the past, gynecologists have conducted a large number of studies to establish a model of predicting suboptimal debulking surgery (SDS) to guide clinical practice, mainly focusing on computed tomography (CT) [10–16], magnetic resonance imaging (MRI) [14, 15, 17, 18], positron emission tomography-CT (PET-CT) [19, 20], ultrasonography [21], laparoscopy [2, 22–27], immunology [28], and so on. These methods have advantages but also disadvantages. Models based on CT, MR, PET-CT, and ultrasonography is highly subjective and has low repeatability, requiring gynecologists to have rich experience and image-reading skills. Laparoscopy is the gold standard for tumor burden assessment, but not the optimal approach considering the physical trauma and economic burden on the patients. Extensive heterogeneity is an important feature of tumors, and it may result in the divergent extent to which the tumor spreads [29–31]. Immunology-based models can demonstrate intratumoral heterogeneity, but it cannot provide information about the tumor burden, adjacent condition of the primary tumor, and implantation metastasis in the abdominal pelvis. The ODS rates reported in previous studies varied widely from 59.6 to 84.5% [13, 15–18]. It may be related to the poor repeatability of the preoperative assessment models besides the technical level of surgical teams. Therefore, a more objective, accurate, and reproducible model that can guide tumor heterogeneity for the preoperative evaluation of the possibility of SDS should be developed.

Machine learning methods have been pervasive in medical image analysis [32, 33]. Radiomics has the characteristics of high-throughput image feature extraction and high repeatability. In several previous studies, the combination of machine learning and radiomics has shown advantages [34–39] for classification, differential diagnosis, evaluation of chemotherapy efficacy, and survival prediction of OC. Thus far, only a few studies have focused on the preoperative evaluation of SDS based on radiomics. Li et al. [20] used a radiomic-clinical model to assess residual disease after PDS, but the radiological feature involved was only characteristic of the ovarian mass. Thus, given the tumor heterogeneity, mechanisms of peritoneal implantation metastasis, and the surgical focus of OC, we intend to develop and validate externally a radiomic–clinical–radiological model based on MRI for preoperative evaluation of SDS, combining radiomic signatures, clinical characteristics, ovarian mass features and its adjacent situation, and abdominal implantation lesion on MR images.

## Materials and methods

### Patients

The Ethics Committee of institutions A and B approved the study protocol, and the need for informed consent was waived owing to the retrospective nature of this study.

**Fig. 1:**
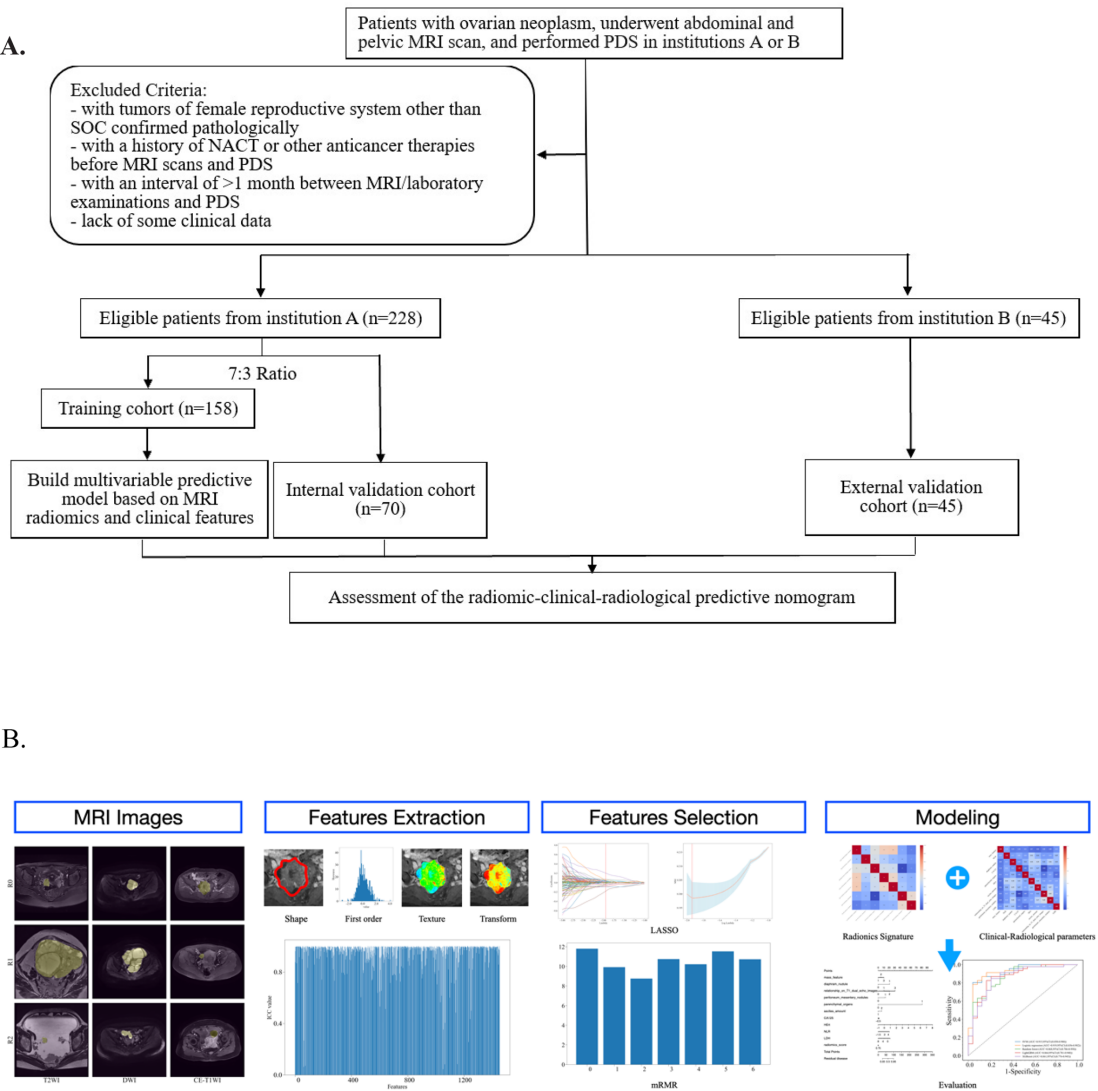
Workflow of our study. **A** Flowchart of our study; **B** Four steps of our predictive model building: image segmentation, features extraction, features selection and dimensionality reduction, modeling and performance evaluation

The workflow of this study is summarized in Fig. 1A. We initially screened 2779 patients with ovarian neoplasm who underwent preoperative abdominal pelvic MR and PDS in institution A from January 2016 to March 2021, by searching the picture archiving and communication system (PACS) of institution A. Clinical data of all patients initially included were recorded, including age, carbohydrate antigen 125 (CA-125), human epididymis protein 4 (HE-4), serum lactate dehydrogenase (LDH) level, neutrophil-to-lymphocyte ratio (NLR), and American Society of Anesthesiologist classification (ASA).

Subsequently, patients were further excluded if they met any of the following exclusion criteria: (1) patients with a history of NACT or other anticancer therapies before MRI scans and PDS, (2) patients with tumors of female reproductive system other than SOC confirmed pathologically, (3) with an interval of > 1 month between MRI/laboratory examinations and PDS, and (4) lack of some clinical data. Finally, a total of 228 patients at institution A were included in this study.

By searching the PACS of institution B, we initially screened 497 patients with ovarian neoplasm from January 2019 to March 2022. The inclusion and exclusion criteria were the same as that in institution A applied to 45 patients from institution B as the external validation cohort.

The outcomes of ODS/SDS were assessed based on postoperative note. According to Li et al. [18], ODS is deemed as R0, whereas SDS is considered R1 or R2 (a residual disease with a maximum diameter of ≥ 1 cm) in this study.

### MRI protocols

In institution A, abdominal pelvic MRI was performed with 3.0 Tesla whole-body MRI system (Signa HDxt, GE Medical Systems, Milwaukee, Wisconsin) using a body phased-array coil that ranged from the top of the diaphragm to the inferior pubic symphysis. All imaging sequences were performed in the axial planes, and the detailed parameters were as follows: T1-dual echo imaging (T1-DEI) (flip angle/time of repetition [TR], 80°/265 ms), slice thickness, 5 mm; gap, 1 mm; field of view, 40 cm; number of excitations (NEX), 0.5; T2-weighted imaging fat-suppressed fast spin-echo (T2FSE) (TR/time of echo [TE], 4260 ms/108.6 ms); diffusion-weighted imaging (DWI) using spin-echo echo-planar imaging (SE-EPI; TR/TE, 5500 ms/63.9 ms), b value, 800 s/mm^2^; and contrast-enhanced T1WI liver acquisition with volume acceleration 3D with fat saturation (LAVA, T1 with contrast enhancement [T1CE]) (TR/TE, 4 ms/2 ms) immediately after the injection of gadopentetate dimeglumine (0.1 mmol/kg body weight, Magnevist; Bayer Schering) injected at a rate of 2–3 mL/s and then repeated at 30, 60, 90, and 120 s during the examination. The scanning parameters of T1CE were as follows: slice thickness, 4 mm; gap 2 mm; matrix, 320 × 224; field of view, 40 cm; and 0.7 excitation.

In institution B, the 1.5 Tesla whole-body MRI system (Signa HDxt, GE Medical Systems) was used, including the same scan range and sequences, and the detailed parameters were as follows: T1-DEI (flip angle/TR, 80°/200 ms), slice thickness, 5 mm; gap, 1 mm; field of view, 40 cm; NEX, 0.75; T2FSE (TR/TE, 2700 ms/68 ms); DWI (TR/TE, 4000 ms/74.8 ms), b value, 800 s/mm^2^; LAVA T1CE (TR/TE, 4 ms/2 ms) immediately after the injection of gadopentetate dimeglumine (0.1 mmol/kg body weight, Magnevist; Bayer Schering) injected at a rate of 2–3 mL/s, and then repeated at 26, 60, 90, and 120 s during the examination. The scanning parameters of T1CE were as follows: slice thickness, 4 mm; gap 0 mm; matrix, 256 × 224; field of view, 40 cm; and 0.7 excitation.

### MRI analysis

MR images were analyzed dependently by two experienced radiologists with > 10 years of experience in gynecologic MRI on PACS workstation, and then any discrepancy was resolved by discussion. The following radiological characteristics were recorded, which were closely related to debulking surgery of SOC: (I) unilateral or bilateral masses; (II) ovarian mass feature (mainly cystic [solid component accounted for less than one-third], mixed solid and cystic [solid component accounted from one to two-thirds], and mainly solid [solid component accounted for more than two-thirds]); (III) relationship between sigmoid colon/rectum and ovarian mass or mass implanted in Douglas’ pouch on MR T1-DEI. To categorize the degree of the relationship, a four-point scale was used: (0) (clear; the boundary between the sigmoid colon/rectum and the ovarian mass is clear, and a hook edge sign is present between the two; (1) (close; disappearance of the hook edge but the shape of the two can be vaguely distinguished); (2) (bridge sign; disappearance of the hook edge and limited adhesion between the two); (3) (fusion; disappearance of the hook edge and the two fused into a block); (IV) diaphragm nodules (nodules implanted below the diaphragm); (V) peritoneum/mesentery nodules (> 1 cm nodules implanted on the peritoneum or mesentery); (VI) metastases of distant parenchymal organs in the abdomen; (VII) retroperitoneal lymph node metastasis in suprarenal aortic level; and (VIII) amount of ascites (small, ascites confined to the pelvis; medium-to-large, ascites beyond the pelvis).

### Tumor segmentation, feature extraction, and repeatability test

Before tumor segmentation, all MRI sequences were resampled to the same voxel size (1 mm × 1 mm × 5 mm) with linear interpolation to ensure the conservation of scales and directions when deriving the 3D features. Z-score normalization was also performed as a preprocessing step for the data to ensure the repeatability of the results and reduce potential effects associated with scan parameters, protocols, scanners, and manufacturers [40].

The 228 patients were randomly divided into a training cohort (n = 158) and an internal validation cohort (n = 70) in a 7:3 ratio. The overview of the image processing–modeling of our workflow is shown in Fig. 1B. ITK-SNAP software was used for tumor segmentation of all ovarian masses (version 3.8.0, http://openiconlibrary.sourceforge.net/). All the regions of interest (ROIs) for each ovarian mass were delineated manually slice-by-slice by two radiologists with more than 10 years of experience in gynecological MRI using T2FSE, DWI, and T1CE, respectively. Any discrepancy was resolved by consensus. For patients with bilateral masses, a set of radiomic features was generated from the larger one.

Subsequently, the open-source Python package Pyradiomics (v. 2.1.1; http://www.radiomics.io/pyradiomics.html) was used to extract 1454 handcrafted radiomic features from each sequence. The handcrafted features mainly include four groups: morphologic features, intensity-based (first-order), texture (second-order), and transformation features. We also selected a subset of the training cohort with 50 typical data to repeatedly delineate the ROIs 1 month later. The intraclass correlation coefficients (ICC) between interobservers and intraobservers are calculated to exclude nonreproducible features below an ICC threshold. In this experiment, features with ICC ≥ 0.90 were considered robust [41].

### Feature selection, radiomic model construction, and assessment

After the repeatability test, three-step feature selection methods were used to minimize overfitting and identify the features that were most effective for classification. First, the t-test was used to analyze whether the means of two classes of samples were significantly different from their distributions. Then, the least absolute shrinkage and selection operator (LASSO) was used to eliminate features with high correlation. The same procedures were applied to the data of the three sequences, leaving 13, 13, and 2 features. Finally, the minimum redundancy–maximum relevance (mRMR) [42] was used to remove redundancy and retain the controllable number of features [43]. The area under the curve (AUC) of the receiver operating characteristic (ROC), sensitivity, and specificity were evaluated in advance by logistic regression to determine the number of features.

Moreover, five machine learning methods (i.e., random forest, logistic regression, K-Nearest Neighbor [KNN], Light Gradient Boosting Machine [LightGBM], and eXtreme Gradient Boosting [XGBoost]) were used to build radiomic signatures to predict SDS [27], which are single sequence for each MRI sequence, dual-sequence, and all-sequence. By comparison, the optimal sequence and the best machine learning method were selected for combination as an optimal radiomic model. The Bonferroni-correction [44] was used to control the chance of making a type-I error (false-positive) for multiple hypothesis testing within sets of five radiomics models and five sequences/sequence combinations, and we set the threshold to determine statistical significance in this phase of analysis to be 0.005 (0.05/10). The predictive powers of all radiomic models were evaluated in the training cohort based on AUC, accuracy, sensitivity, specificity, etc., and validated in the internal and external validation cohorts.

### Radiomic–clinical–radiological model construction and assessment

Univariate logistic regression was used to determine the optimal cut-off values of the numerical variables (age, CA-125, HE4, LDH and NLR), which were converted into categorical variables. Univariate and multivariate analyses were performed for six clinical variables and eight MR variables of the original study design, and independent predictors were identified. Then the clinical–radiological model was established. The weights of the optimal radiomics model and the clinical–radiological model were obtained, and the two models were integrated to finally obtain the radiomic–clinical–radiological model by multivariable logistic regression. The predictive powers of clinical–radiological and the radiomic–clinical–radiological models were evaluated in the training cohort based on AUC, accuracy, sensitivity, specificity, etc., and validated in the internal and external validation cohorts.

### Statistical analysis

Statistical analysis was performed using the Python package scipy (https://scipy.org/), and the radiomic–clinical–radiological model and relative nomogram was developed using the R software (version 3.5.1, http://www.r-project.org/). Differences in clinical and radiological characteristics between the ODS group (R0) and SDS group (R1 + R2) in training cohorts, and the training and internal validation cohorts were assessed using the Student’s t test and the chi-square test for numerical and categorical variables, respectively. For the ROC curves of the models, the DeLong method was used to assess the differences, and P < 0.05 was considered significant.

## Results

### Study population

A total of 228 patients with SOC in institution A were enrolled from 2779 patients who developed ovarian mass according to the selection criteria. The average age of 228 patients with SOC eligible from institution A was 55 years (range 27 ~ 80 years). The clinical and MRI characteristics of these patients are shown in Tables 1 and 2, respectively. No significant differences in the clinical and MRI characteristics were found between the training and internal validation cohorts (*p* > 0.05). Forty-five patients from institution B were included as the external validation cohort, and their clinical and MRI characteristics are shown in Table 3. The average age of 45 patients with SOC eligible from institution B was 54 years (range 30 ~ 74 years).

**Table 1:** Clinical characteristics of patients from institution A

Characteristics	Training cohort, n = 158	Internal validation cohort, n = 70	*P* value
Debulking results			0.980^c^
R0	55	24	
R1 + R2	103	46	
Clinical characteristics			
Age (years)	53.04 ± 9.85	55.03 ± 9.53	0.132^a^
CA125 (U/ml)	974.55 (359.78, 2453.15)	948.9 (318.76, 2920.20)	0.819^b^
HE4 (pmol/L)	359 (146.50, 718.75)	320.16 (162, 703.25)	0.782^b^
LDH (U/L)	219.5 (167.25, 351.50)	210.5 (174.50,314)	0.975^b^
NLR	2.93 (1.95, 5.67)	3.4 (2.12, 4.70)	0.572^b^
ASA			0.060^c^
I–II	32	23	
III–IV	126	47	

Debulking results and ASA are shown as number of patients; age is shown as mean ± standard deviation; CA-125, HE-4, LDH, and NLR are expressed as medians (interquartile ranges); ^a^Two independent samples Student’s t test; ^b^Wilcoxon’s test; ^c^Chi-squared test. R0 = the absence of residual disease; R1 = a residual disease with a maximum diameter of < 1 cm; R2 = a residual disease with a maximum diameter of ≥ 1 cm; *CA-125* carbohydrate antigen 125, *HE-4* human epididymis protein 4, *NLR* neutrophil-to-lymphocyte ratio, *LDH* lactate dehydrogenase, *ASA* American Society of Anesthesiologist classification

**Table 2:** MRI characteristics of patients from institution A

Characteristics	Training cohort, n = 158	Internal validation cohort, n = 70	p
Debulking results			0.980
R0	55	24	
R1 + R2	103	46	
MRI characteristics			
Distribution			0.104
Unilateral	68	39	
Bilateral	90	31	
Mass feature			0.930
Mainly cystic	24	12	
Mixed solid and cystic	47	20	
Mainly solid	87	38	
Relationship between sigmoid colon/rectum and mass on T1-dual echo images			0.854
0	29	13	
1	25	8	
2	63	30	
3	41	19	
Nodules implanted below the diaphragm			1.000
0	114	51	
1	44	19	
Nodules (> 1cm) implanted on the peritoneum or mesentery			0.642
0	40	15	
1	118	55	
Metastases of distant parenchymal organs in the abdomen			1.000
0	147	65	
1	11	5	
Retroperitoneal lymph node metastasis in suprarenal aortic level			0.634
0	130	55	
1	28	15	
Amount of ascites			0.313
None/small	67	24	
Medium-to-large	91	46	

Relationship between sigmoid colon/rectum and mass 0, 1, 2, and 3 represent clear, close, bridge sign, fusion, respectively

R0 = absence of residual disease; R1 = a residual disease with a maximum diameter of < 1 cm; R2 = a residual disease with a maximum diameter of ≥ 1 cm

Debulking results and MRI characteristic data are numerical data, using a chi-square test

**Table 3:** Clinical and MRI characteristics of patients from institution B

Characteristics	External validation cohort, n = 45
Debulking results	
R0	19
R1 + R2	26
Clinical characteristics	
Age (years)	54.29 ± 8.13
CA125 (U/ml)	1000 (404.88, 1655)
HE4 (pmol/L)	558 (196.30, 840.50)
LDH (U/L)	348.2 (226, 523.10)
NLR	3.76 (2.84, 5.51)
ASA	
I–II	7
III–IV	38
MRI characteristic	
Distribution	
Unilateral	24
Bilateral	21
Mass characteristic	
Mainly cystic	5
Mixed solid and cystic	19
Mainly solid	21
Relationship between sigmoid colon/rectum and mass	
0	7
1	9
2	19
3	10
Nodules implanted below the diaphragm	20
Nodules (> 1cm) implanted on the peritoneum or mesentery	34
Metastatic lymph nodes in suprarenal aortic level	7
Metastases of distant parenchymal organs in the abdomen	3
Amount of ascites	
None/Small	21
Medium-to-large	24

Debulking results and MRI characteristic data are numerical data, expressed as absolute numbers

*CA-125* carbohydrate antigen 125, *HE-4 *human epididymis protein 4, *LDH* lactate dehydrogenase, *NLR* neutrophil-to-lymphocyte ratio, *ASA *American Society of Anesthesiologist classification

### Radiomic model construction and internal and external validation

**Fig. 2:**
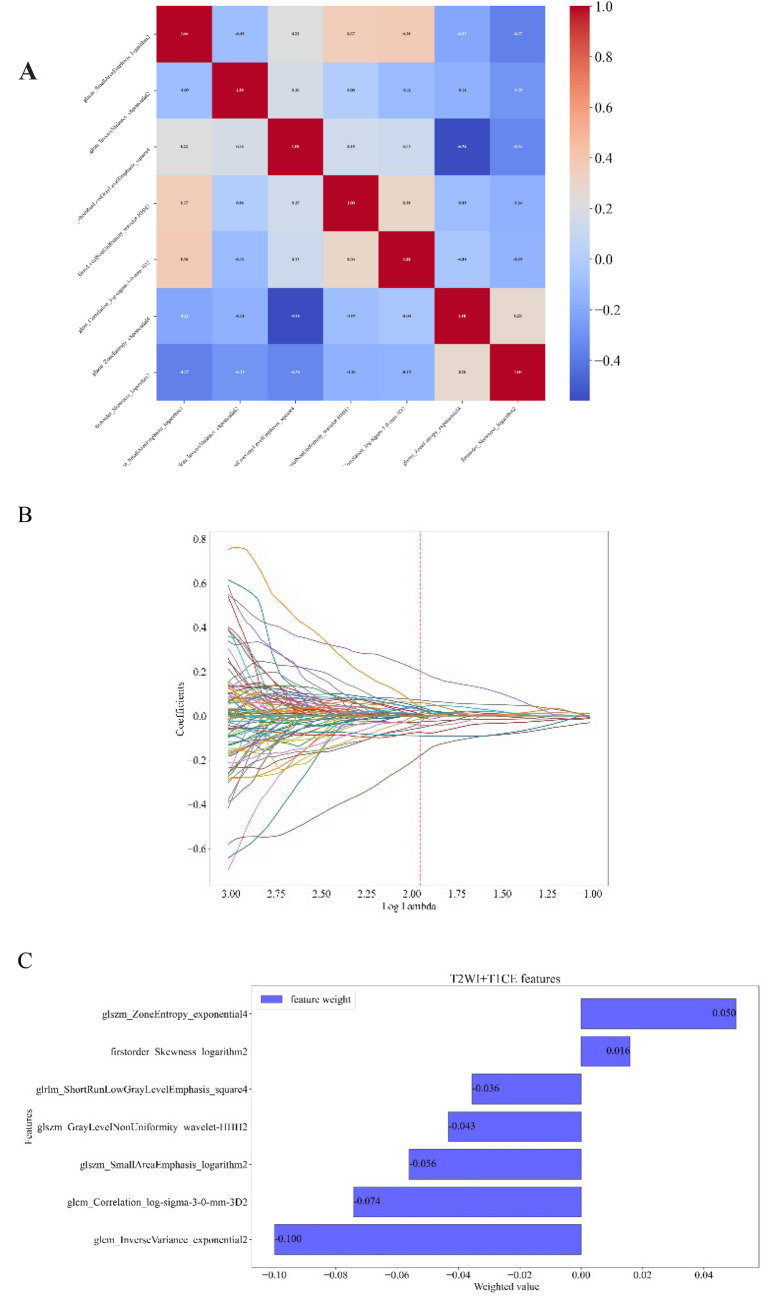
Selection of radiomic features via the Correlation heatmap (T2FSE + T1CE). **A** Linear correlation after feature selection; **B** LASSO coefficient profiles of the 1454 radiomics features. The selected log λ value corresponds to a coefficient of-1.939. **C** The weight coefficient of the seven retained features of dual-sequence (T2FSE + T1CE)

For each MRI sequence (T2FSE, DWI, and T1CE), a total of 1454 features were extracted. By ICC test, 1292 image features were remained. By eliminating high correlation features (t-test and LASSO) and redundancy (m-RMR), five, five, and two features were preserved for the radiomic model construction (Table 4). The random forest model was optimal among the five radiomic models (random forest, logistic regression, KNN, LightGBM, and XGBoost) after Bonferroni–correction (*p* < 0.005). In the internal validation cohort, the AUCs of T2FSE, DWI, T1CE, and combined radiomic model (T2FSE + T1CE and T2FSE + DWI + T1CE) were 0.656, 0.664, 0.700, 0.720, and 0.700, respectively (Table 5). The radiomic model (T2FSE + T1CE) was optimal after Bonferroni–correction (*p* < 0.005). Seven characteristics of this model were retained, as shown in Fig. 2, and the value of log λ is − 1.939.

**Table 4:** Features preserved for radiomics model construction

T2FSE	DWI	T1CE
glszm_SmallAreaEmphasis_logarithm	glszm_SmallAreaEmphasis_logarithm	glrlm_ShortRunLowGrayLevelEmphasis_square
glszm_GrayLevelNonUniformity_wavelet-HHH	glszm_GrayLevelNonUniformity_wavelet-HHH	glszm_ZoneEntropy_exponential
glcm_Correlation_log-sigma-3-0-mm-3D	glcm_Correlation_log-sigma-3-0-mm-3D	
glcm_InverseVariance_exponential	glcm_InverseVariance_exponential	
firstorder_Skewness_logarithm	firstorder_Skewness_logarithm	

*T2FSE* T2-weighted imaging fat-suppressed fast spin-echo, *DWI* diffusion-weighted imaging, *T1CE* T1 with contrast enhancement

**Table 5:** Performance evaluation of each radiomic model

Index	Radiomic models				
	T2FSE	DWI	T1CE	T1CE + T2FSE	T1CE + DWI + T2FSE
Sensitivity	0.674	0.652	0.673	0.630	0.891
Specificity	0.625	0.625	0.667	0.708	0.458
Accuracy	0.657	0.643	0.671	0.657	0.742
AUC	0.656	0.664	0.700	0.720	0.700

*T2FSE* T2-weighted imaging fat-suppressed fast spin-echo, *DWI* diffusion-weighted imaging, *T1CE* T1 with contrast enhancement, *AUC* area under the curve

### Radiomic–clinical–radiological model construction and internal and external validation

The best cut-off values of each numerical variables (age, CA-125, HE4, LDH, NLR) were determined by univariate logistic regression in order to be converted into categorical variables: Age (cut-off value = 57, C-index = 0.687), CA-125 (cut-off value = 918, C-index = 0.723), HE-4 (cut-off value = 276, C-index = 0.742), LDH (cut-off value = 227, C-index = 0.742), C index = 0.651), NLR (cut-off value = 3.98, C-index = 0.657).

**Fig. 3:**
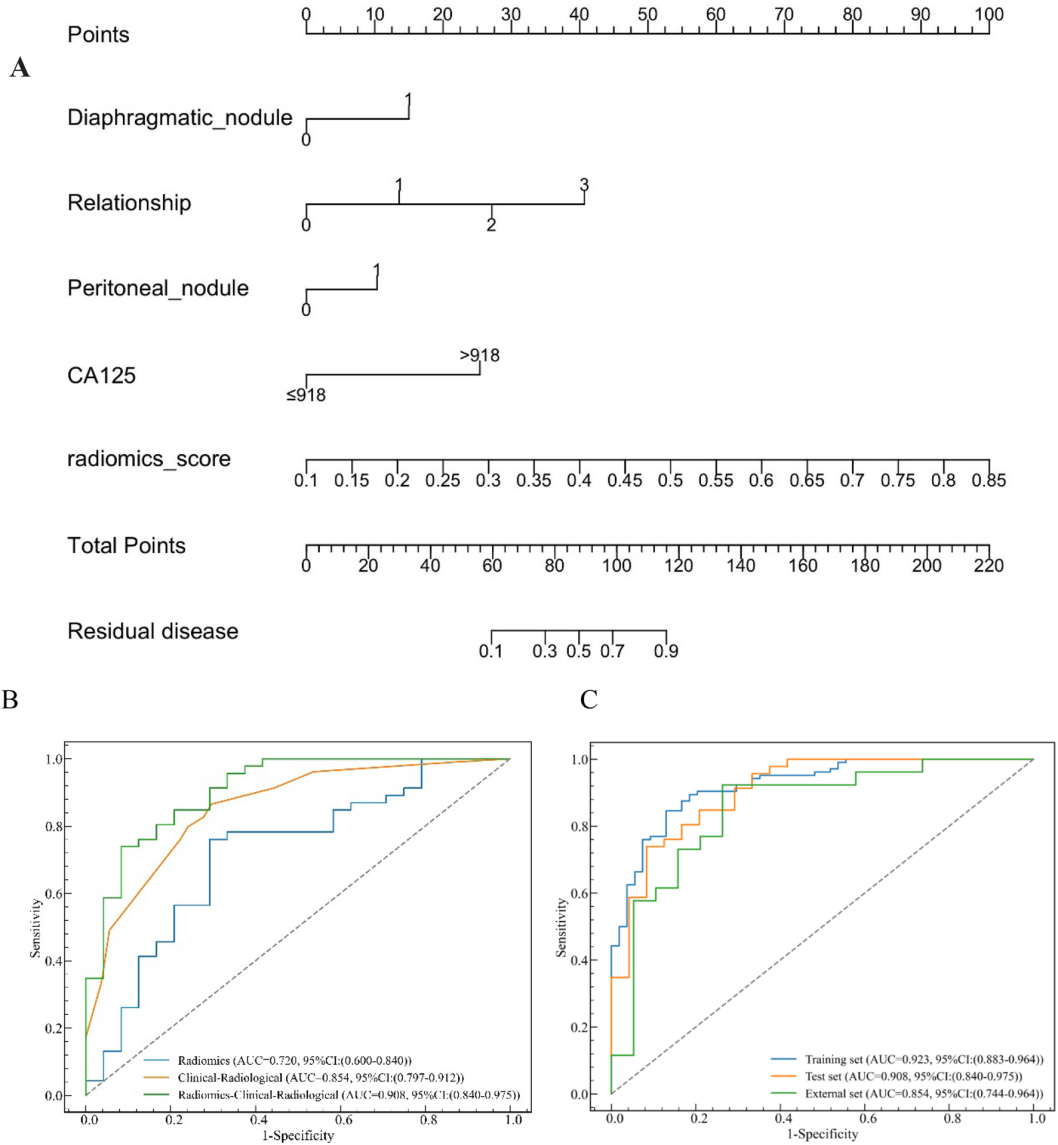
The radiomic–clinical–radiological model and its performance. **A** The radiomic–clinical–radiological model for the training cohort. **B** ROC curves of the radiomic, clinical–radiological, and radiomic–clinical–radiological models for the internal validation cohort. **C** The corresponding ROC curves of radiomic–clinical–radiological model for the three cohorts

**Fig. 4:**
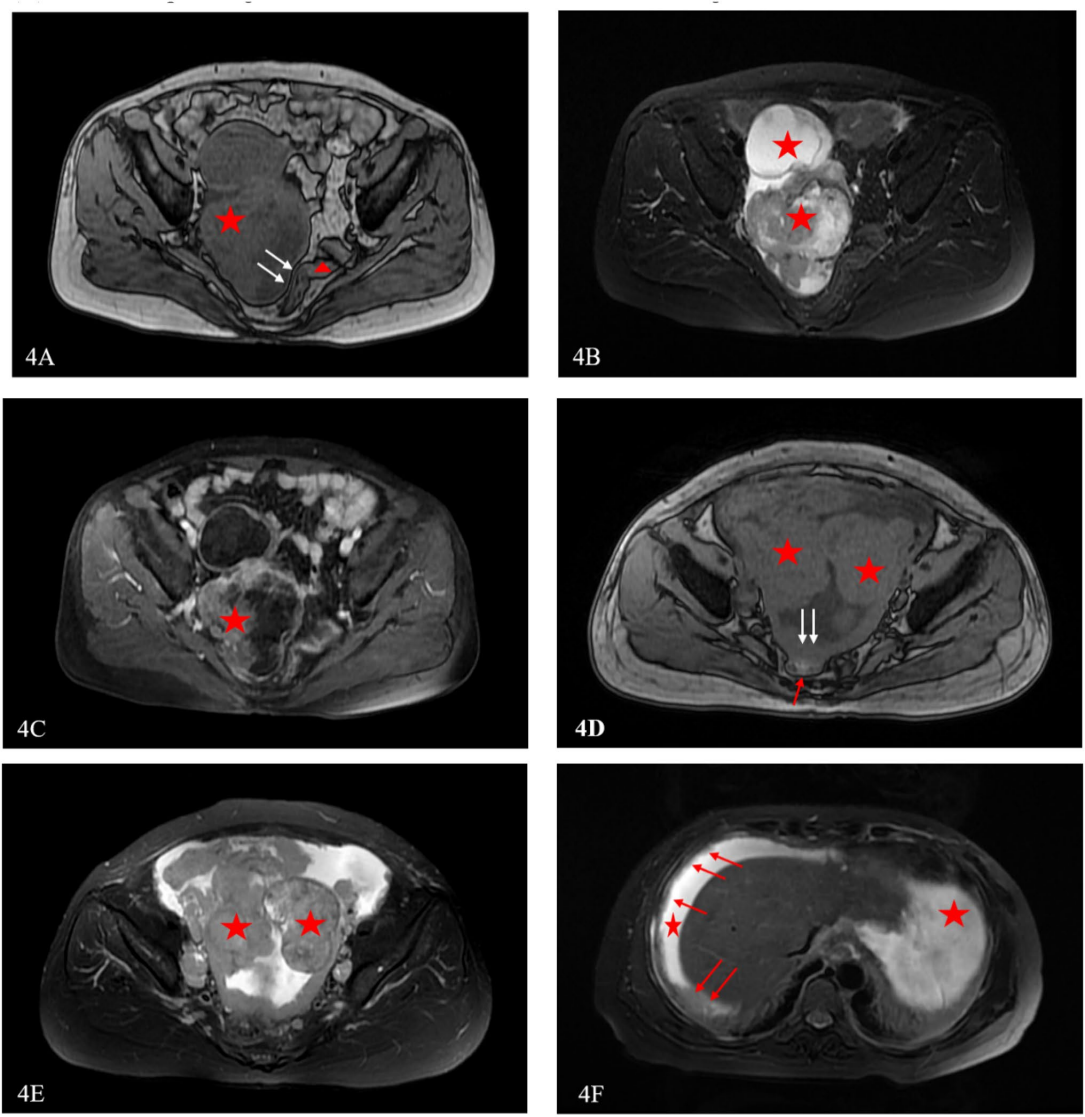
Two cases of MR images representing R0 (**A**–**C**) and R2 (**D**–**F**), respectively. **A**–**C** A 58-year-old woman with high grade SOC (HGSOC), who achieved R0 resection by PDS. **A** Axial T1DEI image shows a mass on right ovary (red star) without invasion (white arrows) of the adjacent rectum (red triangle). **B** Axial T2FSE image shows a cystic-solid mass on the right ovary (red stars). **C** Axial T1CE image shows the solid component of the right ovarian mass was significantly enhanced (red star). **D**–**F** A 49-year-old woman with HGSOC, who achieved R2 resection by PDS. **D** Axial T1-DEI image shows solid mass (red stars) of bilateral ovary. The metastasis implanted on pelvic peritoneal in front of rectum (red arrow) was fused to the rectum as a block (white arrows). **E** Axial T2FSE image shows solid mass (red stars) of bilateral ovary. **F** Axial T2FSE image shows large amount of ascites (red stars), and metastasis implanted on the diaphragm (red arrows)

After univariate and multivariate analysis (Tables 6 and 7), serum CA-125, relationship between sigmoid colon/rectum and ovarian mass or mass implanted in Douglas’ pouch, diaphragm nodules, and peritoneum/mesentery nodules among the clinical and radiological features were considered independent predictors. Then the radiomic–clinical–radiological model was constructed by combining the independent predictors and predictive scores of the optimal radiomic model (Fig. 3). As shown in Fig. 3A, the radiomics score was the most important among the five variables, and the relationship between the sigmoid colon/rectum and ovarian mass or the mass implanted in Douglas’ pouch on MR T1-DEI images was the most important radiological variable. Figure 3B shows the ROC curves for the optimal radiomic, clinical–radiological, and radiomic–clinical–radiological models. As shown in Table 8, among the three cohorts, the radiomic–clinical–radiological model showed remarkable prediction ability with AUCs of 0.923, 0.908, and 0.854, respectively (*P* < 0.05). The AUC value of the radiomic–clinical–radiological model in external cohort was still as high as 0.854 (Fig. 3C). Figure 4 shows the radiological characteristics related to ODS/SDS on MR images.

**Table 6:** Univariate analysis for clinical and MRI features

Clinical and MRI features	OR	95% *CI*	*P* value
Clinical characteristics			
Age			
≤ 57 years	1		
> 57 years	0.97	0.94 – 1.00	0.03
CA-125			
≤ 918 U/ml	1		
> 918 U/ml	0.25	0.100—0.63	< 0.001
HE4			
≤ 276 pmol/L	1		
> 276 pmol/L	4.8	2.64—8.71	< 0.001
LDH			
≤ 227 U/L	1		
> 227 U/L	5.28	2.92—9.54	< 0.001
NLR			
≤ 3.98	1		
> 3.98	0.71	0.61—0.84	< 0.001
ASA			
I–II	1		
III–IV	0.73	0.38—1.42	0.36
MRI characteristics			
Distribution			
Unilateral	1		
Bilateral	0.54	0.31- 0.95	0.03
Mass feature			
Mainly cystic	1		
Mixed solid and cystic	0.42	0.18–0.96	0.04
Mainly solid	0.31	0.14–0.67	< 0.001
Relationship between sigmoid colon/rectum and mass on T1-dual echo images			
0	1		
1	1.2	0.44–3.27	0.73
2	0.12	0.05–0.28	< 0.001
3	0.04	0.01–0.13	< 0.001
Nodules implanted below the diaphragm			
0	1		
1	0.04	0.01–0.16	< 0.001
Nodules (> 1cm) implanted on the peritoneum or mesentery			
0	1		
1	0.08	0.04–0.17	< 0.001
Metastases of distant parenchymal organs in the abdomen			
0	1		
1	0.01	0–0.05	0.99
Retroperitoneal lymph node metastasis in suprarenal aortic level			
0	1		
1	0.25	0.1–0.63	< 0.001
Amount of ascites			
None/Small	1		
Medium-to-large	0.23	0.13–0.41	< 0.001

Chi-squared test, *CA-125* carbohydrate antigen 125, *HE-4* human epididymis protein 4, *NLR* neutrophil-to-lymphocyte ratio, *LDH* lactate dehydrogenase, *ASA* American Society of Anesthesiologist classification, *OR* odds ratio, *CI* confidence interval

**Table 7:** Multivariate analysis for clinical and MRI features

Clinical and MRI features	OR	95% *CI*	*P* value
Clinical characteristics			
CA-125			
≤ 918 U/ml			
> 918 U/ml	0.280	0.100–0.740	0.01
MRI characteristics			
Relationship between sigmoid colon/rectum and mass on T1-dual echo images			
0	1		
1	–	–	–
2	6.022	1.951–20.014	0.002
3	15.985	3.971–75.728	0.0001
Nodules implanted below the diaphragm			
0	1		
1	9.060	2.143–65.060	0.008
Nodules (> 1cm) implanted on the peritoneum or mesentery			
0	1		
1	3.138	1.125–8.955	0.030

Chi-squared test, *CA-125* carbohydrate antigen 125, *OR* odds ratio, *CI* confidence interval

**Table 8:** Performance evaluation of the radiomic, clinical–radiological, and radiomic–clinical–radiological models

Cohorts	Models	Index			
		Sensitivity	Specificity	Accuracy	AUC
Training	Radiomic	0.689	0.891	0.760	0.888
	Clinical–radiological	0.913	0.75	0.857	0.903
	Radiomic–clinical–radiological	0.837	0.870	0.848	0.923
Internal validation	Radiomic	0.630	0.708	0.657	0.720
	Clinical–radiological	0.826	0.722	0.791	0.854
	Radiomic–clinical–radiological	0.848	0.708	0.8	0.908
External validation	Radiomic	0.808	0.579	0.711	0.747
	Clinical–radiological	0.846	0.632	0.756	0.826
	Radiomic–clinical–radiological	0.769	0.737	0.756	0.854

*AUC* area under the curve

## Discussion

Several studies [2, 10–22, 24, 26–28] have focused on establishing reliable models to preoperative accurately assess the likelihood of SDS of OC to avoid surgery at an inappropriate time for patients with high tumor load who cannot achieve ODS. However, the extensive implanted metastases complicate the identification of the relationship of the implanted metastases to abdominal organs and the elimination of all lesions. Solving the problem of how to improve the accuracy and repeatability of the prediction model of SDS remains urgent. Compared with previous studies using CT or MRI to predict residual lesions, a predictive model of SDS based on MRI radiomics was used in our study to extract high-throughput image features unrecognizable by the naked eye through machine learning methods, which ensured the repeatability of the method and reduced the difference of human factors in the evaluation process, thus ensuring the robustness of the predicted results. The results of this study show that the model combined with MRI features related to SDS based on radiomics showed significantly higher predictive performance than that of either radiomic or clinical–radiological model alone. The radiomic–clinical–radiological model performed well in the training, internal, and external cohorts (AUC = 0.923, 0.908, and 0.854, respectively). Our findings are consistent with those of a recent study about residual disease prediction of high grade SOC [18], which also based on MRI radiomics. However, our innovations considered the effects of abdominal metastases which have a vital influence on PDS and verified the research results externally.

Previous studies have inconsistent criteria for ODS of OC. For example, Rizzo et al. defined R0 or RD < 1 cm as ODS [15], whereas Li et al. defined R0 as ODS [18]. Chang et al. demonstrated that for each 10% increase in the proportion of patients undergoing R0, the median survival in the cohort increased significantly by 2.3 months [45] compared with patients with RD < 1 cm. Consequently, the SDS was defined as R1 and R2 in this study. For a decade or so, many previous studies have used CT, MRI, ultrasonography, and other imaging methods to identify implantation metastatic lesions of OC and tried to combine them with some clinical indicators to establish an SDS prediction model, which can help gynecologists evaluate the complex conditions of a patient and make appropriate treatment decisions. However, some shortcomings also existed including the presence of complex indicators, neglect of tumor heterogeneity, low reproducibility, and clinical practicability. Compared with CT and ultrasonography, MRI has high soft tissue contrast and multiparameter and multimode, which can more perfectly reflect the internal characteristics of primary tumor lesions, and is more sensitive to intraperitoneal implantation metastatic lesions [14, 17]. Some studies [29–31] have demonstrated that tumor growth, invasion, and metastases can be predicted from the assessment of primary tumors, such as pancreatic ductal adenocarcinoma and advanced gastric cancer. Radiomics reflects heterogeneity within tumors by high-throughput extraction of image features. In recent years, radiomic studies have demonstrated great research value, which were widely used in prediction research of tumor identification, recurrence, disease-free survival, risk of recurrence, and NACT efficacy. Hence, the above practice and studies have provided theoretical support for our research.

The level of CA-125 and HE-4 of patients were significantly increased in this study. By univariate and multivariate analysis, CA-125 was identified as an important factor affecting the prediction model. Compared with the study by Gu et al. [13], the level of CA-125 in this study was higher (918 vs. 800 U/ml), which may be related to the inclusion of cases. All the cases included in this study were SOC, whereas that of Gu’s [13] study were advanced ovarian cancer, which may contain other pathological types of ovarian cancer other than SOC. The increase of CA-125 level in SOC patients was the highest among all ovarian cancer. CA-125 in this study showed a skewed distribution, with the highest value above 30,000 U/ml. Another important influencing factor of the prediction model in this study is the nodules implanted below the diaphragm. This factor has been recognized by some scholars and incorporated into the prediction model of SDS of ovarian cancer [17, 20]. Although the diaphragm is a muscle-fiber structure, it is weak and is immediately adjacent to the thoracic cavity. Therefore, debulking of nodules implanted below the diaphragm increases the difficulty of PDS.

In the present study, three predictive models have been established. As shown in Table 8, the radiomic–clinical–radiological model performed the best, which is consistent with the conclusion of Li et al. [18]. However, the AUC, accuracy, and sensitivity of this model were 0.923, 0.848, and 0.837, respectively, which are higher than those of Li et al. [18]. As regards methodology, the radiomics is highly repeatable. Therefore, we introduced the model based on radiomics and discarded numerous radiological features that were included in other research models, which require subjective evaluation. The variables included in the nomogram of Li et al. [18] were CA-125, HE-4 and ovarian mass characteristics, while we reserved four independent predictive factors through univariate and multivariate analysis among the six clinical variables and eight MRI variables, namely, relationship between the sigmoid colon/rectum and ovarian mass or mass implanted in Douglas’ pouch, diaphragm nodules, and peritoneum/mesentery nodules. The relationship demonstrated the highest weight among the three radiological variables. To our knowledge, our study is the first to include the relationship between the sigmoid colon/rectum and ovarian mass in the model, and the proportion of adhesion or fusion of which was as high as 81.6% (186 / 228). Douglas’ pouch is the most critical area of implantation metastasis of OC. These lesions are often closely related to the sigmoid colon/rectum, so whether they can be completely cleared is not only related to how close the relationship above, but also has a great relationship with whether the patient agrees to partial rectum resection and colostomy when lesion completely removal is difficult. Whether the tumor implanted in Douglas’ pouch can be cut off is crucial to the outcomes of debulking. Besides, a dark border called hook edge effect is found around the organs surrounded by fat on MR T1-DEI. This sign can intuitively indicate the integrity of the margin of the organs in the abdominal pelvis, which is rich in fat signals. It also demonstrated the boundary of the mass and the adjacent organs in the abdominal pelvis. We took this advantage to evaluate the relationships mentioned above. To our knowledge, this is also the first attempt to discern the relationship between the sigmoid colon/rectum and ovarian mass using MR T1-DEI and to include this relationship in a model.

Compared with the dual-sequence radiomic model (T2FSE + T1CE), the all-sequence radiomic model (T2FSE + DWI + T1CE) has four more features: contrast, informational measure of correlation 2, difference variance, and run entropy. The first two features belong to the gray-level co-occurrence matrix feature, the third belongs to the gray-level co-occurrence matrix feature, and the fourth belongs to the gray-level correlation matrix. However, the AUC of the radiomic model (T2FSE + DWI + T1CE) was lower than that of the radiomic model (T2FSE + T1CE) in the present study (0.700 vs. 0.720), and the difference demonstrated significant (*P* < 0.005). Perhaps the contribution of DWI to the model is not positive. Traditional machine learning classifiers such as random forest, logistic regression, and KNN are implemented in Python using the scikit-learn package. Random forest was the optimal radiomics model after Bonferroni–correction (*P* < 0.005) in our study. Therefore, we chose random forest and the dual–sequence (T2FSE + T1CE) as the optimal radiomic model integrating clinical and radiological features to establish a radiomic–clinical–radiological model. The AUCs of the radiomic–clinical–radiological model in the training, internal cohort, and external cohort were 0.923, 0.908, and 0.854, respectively. The AUC value is significantly higher than the research model (training cohort 0.815, internal validation cohort 0.803) of Li et al. (18). Notably, the present study is the first to include an external validation cohort in the radiomic–clinical–radiological model to predict SDS for patients with SOC.

Nevertheless, this study has several limitations. First, the number of patients in the external validation cohort was small. Second, owing to the retrospective nature of this study, there may be bias introduced due to some underlying undetected factors. Third, A small increase in performance (Table 8) was found for the radiomic–clinical–radiological models with respect to the clinical–radiological model in the internal test set, while the performances of the two models were very similar on the external test. Finally, low sensitivity of detection of tiny tumor deposits, like tiny and miliary metastasis in small bowel mesentery was consider a disadvantage of MRI, which requires the technical innovation of MRI. Therefore, this study is sustainable. In the future, we will continue to expand the sample size for prospective studies, explore a clinical-radiomics model to see if radiomics features can replace human evaluation, introduce new surgical technologies and even genomics to explore the influencing factors of SDS.

## Conclusion

Our findings demonstrate an overall algorithm reproducibility of the radiomic–clinical–radiological model by features with good reproducibility. Therefore, the radiomic–clinical–radiological model is helpful for predicting SDS in patients with SOC, which may help create individualized treatment programs, and improve the prognosis of patients.
